# Suicide Among Medical Students and Residents in Iran: Potential Causes and Solutions

**DOI:** 10.34172/aim.2023.10

**Published:** 2023-01-01

**Authors:** Soodeh Jahangiri, Fatemeh Shaygani, Milad Ahmadi Marzaleh

**Affiliations:** ^1^Endocrine and Metabolism Research Institute, Iran University of Medical Sciences, Tehran, Iran; ^2^Student Research Committee, Shiraz University of Medical Sciences, Shiraz, Iran; ^3^Department of Health in Disasters and Emergencies, School of Health Management and Information Sciences, Shiraz University of Medical Sciences, Shiraz, Iran

## Dear Editor,

 According to the most recent investigation into the global burden of disease, suicide attempts account for 1.4% of deaths,^1^ and the suicide rate in Iran was estimated to be 9.9 per 100 000 in 2017.^2^ Physician suicide, especially among medical students and residents, is an alarming issue. Why aren’t the caretakers being taken care of?

 Recent news of residents’ suicide in Iran prompted us to state some possible causes of suicide among Iranian medical students and residents; we also suggest beneficial solutions, hoping that health policymakers design interventional strategies to prevent such tragedies in the future.

## Causes

 Numerous factors may be the cause of this unpleasant event. Undergraduate medical students as well as residents who spend long working shifts in hospitals often experience many inconveniences, such as massive physical and emotional pressure caused by work as well as peers, senior colleagues, and attending. First-year residents in Iran have shifts every other day, which means they are working 36 hours out of every 48 hours. This, of course, varies across different specialties. Despite heavy workload, rules regarding residents’ vacations in Iran’s health system are not applied because of lack of sufficient staff to replace them or of superiors’ disapproval. Along with that, in teaching hospitals, seniors mostly look down upon their junior doctors, and lack of cooperativeness and co-workers’ support leads to emotional exhaustion and eventual burnout.^3^

 A low salary is potentially an important discouraging factor. The average monthly income for residents in Iran is about 200–260 US dollars. This hardly makes up for the expenses of living not only as a couple but also alone. Lack of health insurance coverage is another factor for most residents since they often cannot afford insurance fees. Another challenge is that even when struggling, physicians may avoid seeking professional help due to stigma. Some may think reaching out for help means they are not efficient or may presume that others might consider them incompetent. Many physicians believe they have the required knowledge to treat themselves and avoid visiting a psychiatrist or attending psychotherapy sessions. Lack of peer support is also present. Many individuals will not take their colleagues seriously if they openly talk about their mental health.^4^ Official counseling institutions exist in most universities of medical sciences and they offer free therapy sessions to students, but they do not actively address the issues students and residents are facing.

 It should be noted that during the recent COVID-19 pandemic, a tremendous burden was placed upon healthcare providers, particularly residents. Initial insufficient data about the virus and shortage of personal protective equipment, long shifts in high-risk environments, fear of losing their own or their loved ones’ lives, spending months away from families, and dealing with a high rate of patients’ deaths are factors that lead to stressful conditions (Table 1).

**Table 1:** A Summary of the Causes and Solutions of Medical Students and Residents’ Suicides in Iran

**Causes**	**Solutions**
Work load pressures Almost no vacation	Reassess working hours to reduce doctors' exhaustion Applying vacation rules Assigning a substitute team
Pressure by peers, senior colleagues and attending lack of co-worker support	Developing a friendly work environment and a cooperative and respectful relationship within medical hierarchy
Stigma and false beliefs	Educational and monitoring programs focusing on psychological health Suicide-survivor programs for co-workers mandatory routine psychological evaluations
Low salary No health insurance	Financial support Providing health insurance

## Solutions

 Since this is a multifactorial matter, a multi-disciplinary approach should be taken to overcome it. Developing a friendly work environment and a cooperative and respectful relationship within the medical hierarchy is important.^5^ Authorities should also reassess working hours to reduce residents’ exhaustion, and their vacation rules must be applied; assigning one or two residents as a substitute team is beneficial in this regard. Residents should also receive financial support and an increased salary, as well as health insurance. A study showed that although fatigue is the main reason for job dissatisfaction, salary is mostly associated with more satisfied employees.^6^ Therefore, resolving money issues may be as important as modifying workload.

 To decrease social stigma, educational and monitoring programs should be organized for medical students and residents that focus on their psychological health and coping skills. Depressive thoughts, burnout, and suicidal ideation are not readily recognizable, so authorities must provide mandatory routine psychological evaluations performed by specialists for risk assessment, monthly questionnaires, and free psychotherapy sessions. Apart from providing psychological support especially during the COVID-19 pandemic, paying residents’ overdue and over shift salaries is the least that should be done (Table 1).

## Conclusion

 The support system should warn physicians that medication alone will not solve every problem and many require long-term psychotherapy, especially those suffering from psychiatric disorders. Universities must be aware of the consequences of these suicides, as well. They should provide exclusive suicide-survivor programs and assess the friends, families, and co-workers of deceased individuals; all of them may be greatly affected.
